# Pulmonary Hypertension in Cardiac Surgery

**DOI:** 10.2174/157340310790231671

**Published:** 2010-02

**Authors:** André Denault, Alain Deschamps, Jean-Claude Tardif, Jean Lambert, Louis Perrault

**Affiliations:** Montreal Heart Institute and Université de Montréal, Montreal, Quebec, Canada

**Keywords:** Cardiac surgery, pulmonary hypertension, pharmacological therapy.

## Abstract

Pulmonary hypertension is an important prognostic factor in cardiac surgery associated with increased morbidity and mortality. With the aging population and the associated increase severity of illness, the prevalence of pulmonary hypertension in cardiac surgical patients will increase. In this review, the definition of pulmonary hypertension, the mechanisms and its relationship to right ventricular dysfunction will be presented. Finally, pharmacological and non-pharmacological therapeutic and preventive approaches will be presented.

## 1. DEFINITION OF PULMONARY HYPERTENSION

There are several hemodynamic parameters that are used in defining pulmonary hypertension (PHT) (Table **1**) [1]. Several of these definitions have been used in various studies. In cardiac surgery, we obtain information on PHT before the procedure and this is usually from an awake patient. This preoperative information is either acquired through preoperative catheterization or, more frequently, estimated *via* transthoracic echocardiography by using the Bernoulli’s equation. In the presence of tricuspid regurgitation, as shown in Fig. (**1**), [2] the simplified Bernoulli’s equation will give an estimation of the pressure gradient across the tricuspid valve. This pressure gradient is equal to the difference between the systolic pressure of the right ventricle (RV) and the right atrium (RA). Therefore, knowledge (or estimation) of the right atrial pressure allows the estimation of the right ventricular systolic pressure. In the absence of right ventricular outflow tract obstruction (RVOTO) or pulmonic valve stenosis, this value will be an estimation of the systolic pulmonary artery pressure (SPAP).

**Fig. (2):**
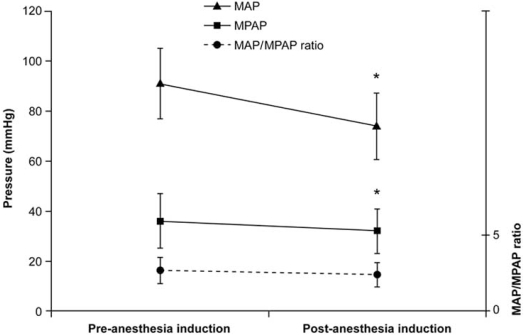
Change in MAP, MPAP, and the MAP/MPAP ratio after the induction of anesthesia in 32 patients with preoperative pulmonary hypertension. No significant change in the MAP/MPAP ratio was observed (*p < 0.05). (MAP: mean arterial pressure, MPAP: mean pulmonary artery pressure) [3].

**Fig. (3):**
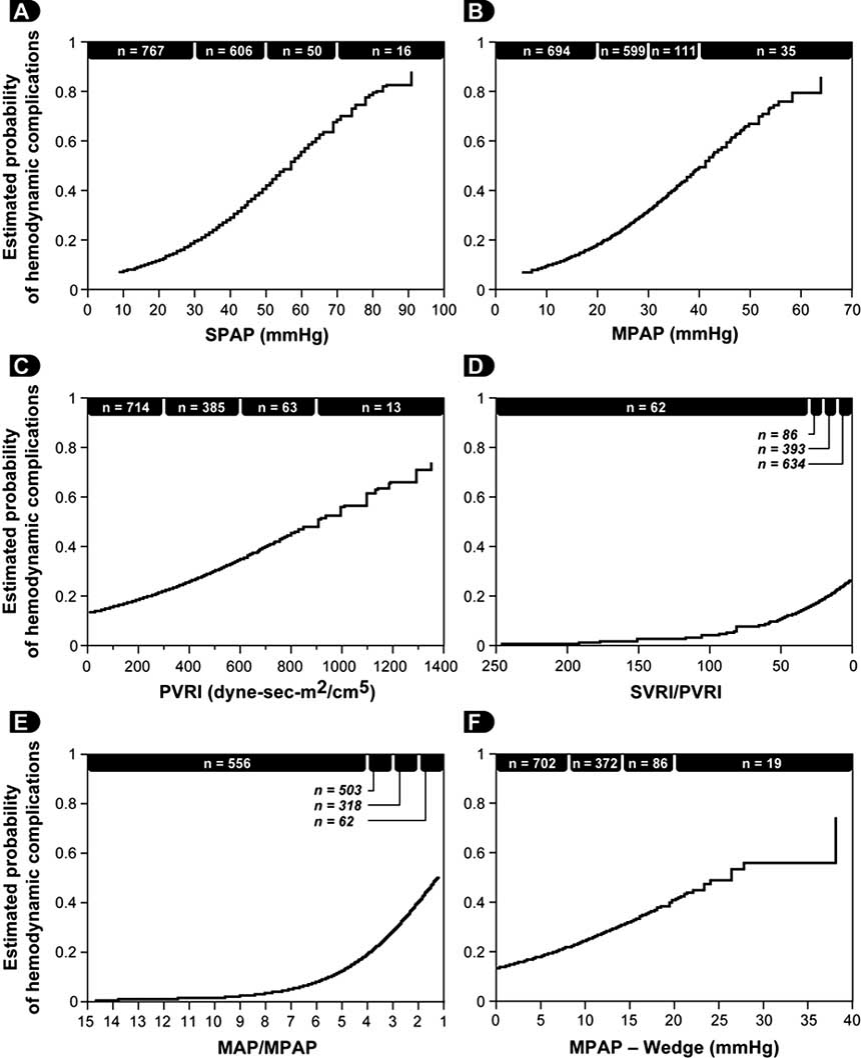
Relationship between the estimated probability of hemodynamic complications and variables used in the evaluation of pulmonary hypertension: (**A**) systolic pulmonary artery pressure (SPAP), (**B**) MPAP, (C) PVRI, (**D**) the ratio of SVRI to PVRI, (**E**) the MAP/MPAP ratio, and (**F**) the transpulmonary gradient defined as MPAP minus pulmonary artery occlusion pressure (PAOP). For easier comparison, the scale of the x axis of the SVRI/PVRI and the MAP/MPAP are inverted. (*n* = number of patients). (MAP: mean arterial pressure, MPAP: mean pulmonary artery pressure, PVRI: indexed pulmonary vascular resistance, SVRI: indexed systemic vascular resistance) [3].

**Fig. (4):**
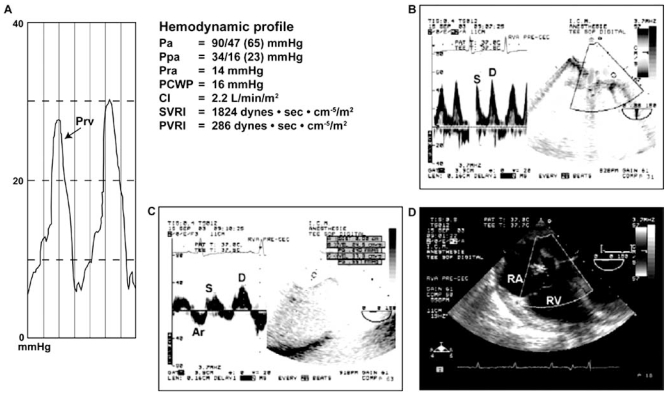
Hemodynamic and transesophageal echocardiographic evaluation of a 46-year-old woman scheduled for aortic valve surgery. Despite a normal pulmonary artery pressure of 34/16 mmHg and PVRI at 286 dyn·s·cm-5·m-2, this patient had an abnormal right ventricular diastolic filling pressure waveform characterized by a rapid upstroke (**A**) and reduced systolic (S) to diastolic (D) pulmonary (**B**) and hepatic (**C**) venous flow consistent with left and right ventricular diastolic dysfunction. In addition, a dilated right atrium and ventricle were present without significant tricuspid regurgitation in a mid-esophageal right ventricular view (**D**). The MAP/MPAP ratio was 65/23 or 2.8. (CI: cardiac index, Pa: arterial pressure, PCWP: pulmonary capillary wedge pressure, Ppa: pulmonary arterial pressure, Pra: right atrial pressure, Prv: right ventricular pressure, PVRI: pulmonary vascular resistance index, RA: right atrium, RV: right ventricle, SVRI: systemic vascular resistance index) [3].

Following the induction of general anesthesia, a reduction of both the systemic and the pulmonary artery pressures will be observed. Consequently, absolute values of SPAP used in defining PHT will tend to underestimate its severity. In 2006, we addressed this issue and published a study involving 1557 patients who underwent cardiac surgery [3]. We first demonstrated that the induction of general anesthesia in 32 patients was associated with a significant reduction in mean arterial pressure (MAP), mean pulmonary artery pressure (MPAP) but the MAP/MPAP ratio did not change (Fig. **2**). Therefore, this ratio (normal value > 4) seems to be a very robust estimator of the severity of PHT. To demonstrate the utility of the MAP/MPAP ratio, we compared it to the other hemodynamic parameters listed in Table **1** in 1439 patients undergoing cardiac surgery after the induction of general anesthesia but before cardiopulmonary bypass (CPB). We observed that the MAP/MPAP ratio behaved similarly to the other hemodynamic parameters (Fig. **3**), and had the highest receiver operating curve value to predict hemodynamic complications after cardiac surgery. The hemodynamic complications were defined as postoperative death or requirement for an intra-aortic balloon pump, cardiac arrest and vasoactive support for more than 24 hours. Finally, using transesophageal echocardiography (TEE), we can confirm that the presence of an abnormal MAP/MPAP ratio is almost invariably associated with abnormal systolic or diastolic cardiac function (Fig. **4**) [3]. This concept of using the relative instead of absolute value of PHT indices is currently used in congenital cardiology [4, 5].

Finally, PHT is typically classified as capillary, precapillary or post-capillary, depending on the site where the cause of PHT is present. The 2003 World Symposium on PHT proposed a classification based on 5 groups: 1-Pulmonary arterial hypertension, 2-PHT secondary to left heart disease, 3-PHT secondary to lung disease and/or hypoxia, 4-PHT secondary to thrombotic and/or embolic disease and 5-A miscellaneous category [6]. In cardiac surgery, it is typically post-capillary or group 2 because the cause of PHT is of cardiac origin and consequently localized after the pulmonary capillary. This is confirmed using pulmonary artery catheterization during which the diastolic pulmonary artery pressure is equal to the pulmonary artery occlusion pressure (PAOP). In a situation where the diastolic pulmonary artery pressure (DPAP) is significantly higher than the PAOP in the absence of tachycardia, a capillary or pre-capillary cause could be sought [1].

In summary, PHT in cardiac surgery should be carefully defined. It is generally a post-capillary PHT. In awake patients, the absolute values have been used and correlated with outcome. However, in patients under general anesthesia, the relative value seems to be more appropriate.

## 2. PULMONARY HYPERTENSION IN CARDIAC SURGERY: MECHANISM AND ETIOLOGY

The mechanism of PHT in cardiac surgery is complex and can result from several mechanisms acting alone or in combination. These mechanisms can be present before the operation, secondary for instance from valvular heart disease. The cause of PHT can appear after CPB from mechanical failure or from the pulmonary reperfusion syndrome. Finally, PHT can be present or persist postoperatively secondary for instance from/to a mitral or aortic patient-prosthesis-mismatch (PPM). Fig. (**5**) focuses on the role of PHT in cardiac surgery [7] based on current literature, our research findings and our experience of this population.

### 2.1. Review of the Factors Involved in Pulmonary Hypertension in Cardiac Surgery

The 6 most important causes of PHT in cardiac surgery factors are illustrated in Fig. (**5**).

**Fig. (6):**
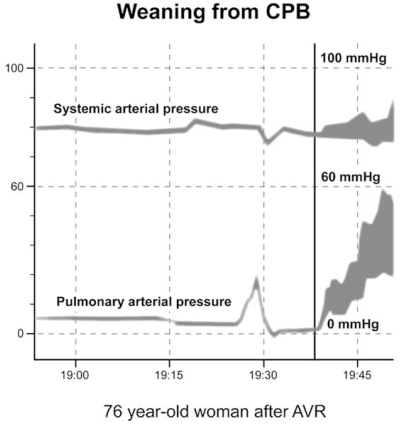
Unexpected pulmonary hypertension upon weaning from cardiopulmonary bypass (CPB) in a 76-year-old woman after aortic valve replacement (AVR). The CPB duration was 71 minutes. A significant increase in pulmonary arterial pressure in relation to the systemic arterial pressure was observed as the patient was weaned from CPB. No mechanical causes were found.

**Fig. (7):**
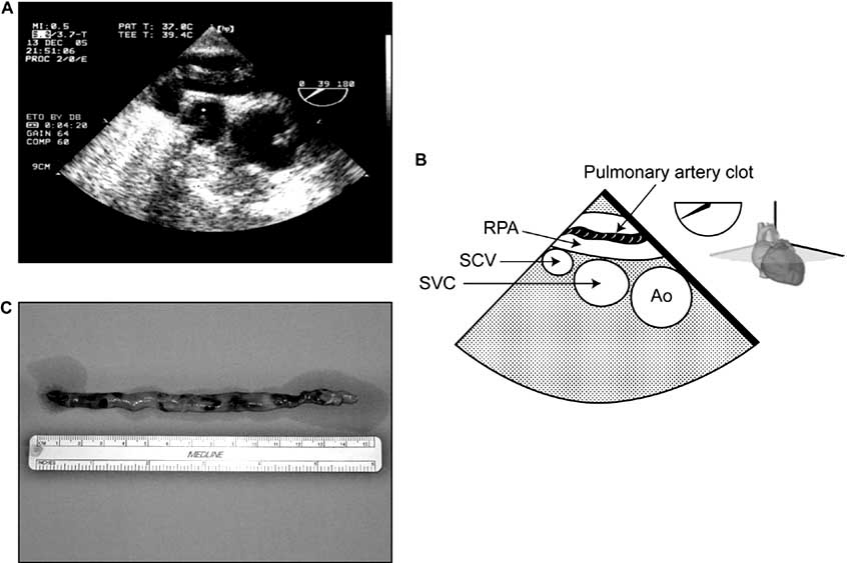
Pulmonary embolism immediately after coronary revascularization. This patient was hospitalized and waiting for more than a week before the procedure could take place. At the end of the procedure while she was transferred in her bed, she became hemodynamically unstable. Immediate transesophageal echocardiographic exam was performed and showed the appearance of a clot in the right pulmonary artery (**A-B**). She was brought back to the operating room for urgent embolectomy and a clot was removed (**C**). She was discharged from the hospital in good condition. (Ao: aorta, RPA: right pulmonar artery, SCV: subclavian vein, SVC: superior vena cava) (Courtesy of Dr. David Braco and Dr. Nicolas Noiseux).

**Fig. (8):**
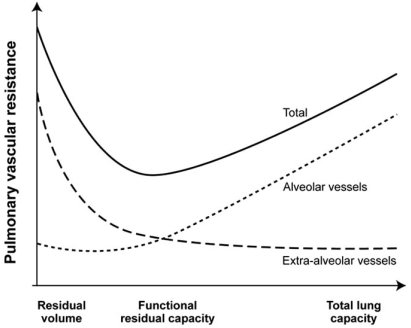
Relationship between lung volume and pulmonary vascular resistance (PVR). PVR is minimal at functional residual capacity (FRC) and increased at large or total lung capacity (TLC) and small lung volumes residual volume (RV) decreases. The differential effect on intraand extra-alveolar vessels accounts for the U-shaped relationship of PVR and lung volume. (Adapted from Fischer *et al*. [18]).

1. Left ventricular systolic or diastolic dysfunction and mitral or valvular disease either pre- or postoperative are the most common causes of PHT in cardiac surgery. Aortic PPM through a reduction in coronary reserve could also contribute to postoperative PHT [8].
2. During cardiac surgery, the extent of the systemic inflammatory response, the pulmonary reperfusion syndrome and the need for blood transfusions may exacerbate PHT (Fig. **6**) [9, 10]. The mechanism of pulmonary damage during extracorporeal circulation is thought to be mainly triggered by 1) release of cytokines [11] through endotoxin production, 2) complement activation and 3) ischemia reperfusion injury [12, 13]. This leads to the production of free radicals, endothelin and prostacyclin derivatives with nitric oxide inhibition [12].
3. The administration of protamine can induce catastrophic pulmonary vasoconstriction in up to 1.8% of patients [14]. Protamine can also activate complement and, when given at the end of CPB, can induce PHT associated with adverse hemodynamic responses ranging from minor perturbations to cardiovascular collapse. Three types have been described: systemic hypotension, anaphylactoid reaction and catastrophic PHT [15]. The mechanism of PHT with protamine is thought to occur through an imbalance of vasoconstrictors and vasodilators which leads a reduction in the release of nitric oxide (NO) from the pulmonary vasculature [15].
4. Mitral PPM is another recently described cause of residual postoperative PHT. Magne *et al.*[16] studied 929 patients who underwent mitral valve replacement (MVR) and followed them up for 15 years. Mitral valve PPM was defined as not clinically significant if > 1.2 cm²/m², as moderate if > 0.9 and ≤1.2 cm²/m², and as severe if ≤0.9 cm²/m². The prevalence of moderate PPM was 69% and that of severe PPM was 9%. Severe PPM was found to be associated with residual PHT and a 3-fold increase in postoperative mortality after adjustment for other risk factors. This new and relevant information is currently absent from the majority of the studies dealing with predictors of survival in mitral valvular surgery.
5. Hypoxia, hypercarbia and pulmonary embolism are other causes of PHT. They can appear before, during or after CPB. For instance, PHT can cause RV dysfunction, which will lead to an increase in right atrial pressure. This can lead to the opening of a patent foramen ovale (PFO), which is present in 20-30% of the general population [17]. The consequence of the opening of a PFO would be a right-to-left shunt. This would increase the severity of hypoxia and lead to an exacerbation of PHT. Pulmonary vessels constrict with hypoxia (Euler-Liljestrand reflex) and relax in the presence of hyperoxia 	[18]. Hypercarbia can occur particularly if acute lung injury occurs during or after the procedure. The increase in PCO_2_ will increase PHT through vasoconstriction. Finally, although pulmonary embolism is rare in the immediate postoperative period, they can occur particularly in patients with predisposing factors (Fig. **7**).
6. Lung volumes have a differential effect on intra- and extra-alveolar vessels, which accounts for the unique U-shaped relationship between lung volume and pulmonary vascular resistance (PVR). PVR is minimal at functional residual capacity and increased at large and small lung volumes (Fig. **8**). Clinically, this may be observed when hyperinflation of the lungs greatly increases PVR [18]. Application of high levels of positive end-expiratory pressure (PEEP) may narrow the capillaries in the well ventilated lung areas and divert flow to less well ventilated or non-ventilated areas, potentially leading to hypoxia. An increase in cardiac output distends open vessels and may recruit previously closed vessels, decreasing PVR. Regional blood flow to lung is also influenced by gravity; pulmonary blood flow is greater in the dependant areas of the lung. In addition, increase in intrathoracic pressure will be transmitted to the surrounding cardiac pressure and contribute to elevate pulmonary artery pressure. Mechanical compression of pulmonary vessels can be caused by hemothoraces or tension pneumothoraces.

Finally, multiple molecular pathways are important for the regulation of PVR. These include the nitric oxide, prostacyclin, endothelin-1 and serotonin pathways [19]. Nitric oxide and prostacyclin are endogenous vasodilators produced in the pulmonary vascular endothelium. Endothelin-1 is an endogenous vasoconstrictor peptide secreted by the vascular endothelium and plays a role in pulmonary vasoconstriction and vascular smooth muscle proliferation [20]. The neurotransmitter serotonin and the serotonin receptor transporter have also been implicated in the regulation of pulmonary vascular tone. An imbalance in these pathways may lead to vasoconstriction and vascular remodelling, potentially leading to progressive pulmonary vascular disease.

The most dreadful consequence of PHT is the increase in RV afterload and RV dysfunction; this issue will be addressed here.

### 2.2 Right Ventricular Dysfunction

There is growing evidence that morbidity and mortality associated with PHT are dependent on RV adaptation to disease rather than on the absolute value of pulmonary arterial pressure [21-25]. In studies addressing hemodynamic variables and survival in idiopathic pulmonary arterial hypertension, high mean right atrial pressures and low cardiac output (CO) were consistently associated with poorer survival when contrasted with pulmonary arterial pressure alone, which was only moderately related to outcome [21, 26].

The importance of RV function in cardiac surgery has been demonstrated in a variety of clinical settings such as high risk coronary or valvular heart disease, congenital heart disease, heart transplantation, in patients requiring mechanical assist devices and in the unstable postoperative patient (Table **2**) [25]. However, most of the evidence that supports the importance of RV function is based on retrospective or small prospective studies. To date, parameters of RV function have not been included in large scale risk stratification models and therefore their incremental value to the Parsonnet Score or the EuroSCORE have not been well established [27-30]. A recent panel from the National Institute of Health (NIH) has stressed the importance of research in the understanding of RV failure [24].

#### 2.2.1. Before the Procedure

In patients presenting with severe aortic stenosis, Boldt *et al.* have demonstrated that preoperative RV dysfunction was associated with a greater requirement of postoperative inotropic support [31]. In a retrospective study of patients undergoing mitral and mitral-aortic valvular surgery, Pinzani *et al*. demonstrated that preoperative RV failure was associated with perioperative mortality. In this same study, postoperative RV failure was the most important independent predictor of late survival [32]. In a small prospective study of 14 patients with severe non-ischemic mitral regurgitation and high risk descriptors (LV ejection (LVEF) ≤ 45% or RV ejection fraction (RVEF) ≤ 20%), Wencker *et al*. found that preoperative RVEF≤ 20% predicted late postoperative deaths [33]. In patients under-going coronary artery surgery, Maslow *et al.* [34] showed than RV dysfunction defined by a RV fractional area change (RVFAC) less than 35% in the context of severe LV systolic dysfunction (LVEF ≤ 25%) and non-emergent coronary artery bypass surgery was associated with an increased risk of postoperative morbidity and mortality. In their retrospective study (*n*=41), patients with RV dysfunction had a higher prevalence of diabetes mellitus and renal disease as well as a higher incidence of postoperative inotropic or mechanical support, longer intensive care unit and hospital stay and a decreased short term and long term survival.

To further assess the value of RV function relative/-compared to other validated risk factors in open valvular heart surgery, we recently published our experience related to 50 patients undergoing valvular surgery [35]. We confirmed that, in patients with a RV myocardial performance index (RVMPI) above 50% (*n*=20), the number of patients with DSB (difficult separation from bypass) (16/20 (80%) *vs.* 6/30 (20%), *p*<0.0001) and the end-point of mortality of postoperative heart failure (14/20 (74%) *vs.* 3/30 (10%), *p*<0.0001) were significantly higher. On a multivariate analysis, among all other demographic, hemodynamic and echocardiographic variables, the RVMPI was the only independent predictor of heart failure and mortality (OR: 25.20, 95%; CI 5.24-121.15, *p*<0.0001).

#### 2.2.2. After the Procedure

The presence of RV failure after CPB is associated with a mortality rate ranging from 44% to 86% [36]. The incidence of post-cardiotomy acute refractory RV failure ranges from 0.04 to 0.1%. Acute refractory RV failure has also been reported in 2-3% patients after a heart transplant and in almost 20-30% patients who receive a left ventricular assist device support with a reported initial salvage rate is only 25-30% [10].

## 3. IMPORTANCE AND IMPACT OF PULMONARY HYPERTENSION IN CARDIAC SURGERY

PHT present before any cardiac procedure is associated with increased morbidity and mortality [27,37-40]. However, the presence of PHT is not routinely reported to the surgeon. This can be explained by the use of preoperative risk stratification models in cardiac surgery, in which only 4/19 models used PHT as a risk factor [41]. Interestingly, the EuroSCORE model, which had the highest discriminatory, is one of the models in which PHT is included. In a study that included 4351 CABG patients operated in Sweden, the receiver operating characteristics (ROC) of EuroSCORE model was 0.86 and 0.75 for the 30-day and one year mortality, respectively.

Using our database in 1999, the mean preoperative systolic pulmonary artery pressure (SPAP) was 31±10 mmHg. Elevated SPAP above 30 mmHg were present mostly in mitral valve replacement (*n*=80, 40±14 mmHg), followed by combined CABG and valve (*n*=126, 36±13 mmHg), multiple valves (*n*=60, 36±16 mmHg) and heart transplantation (*n*=6, 36±14 mmHg). A total of 605 patients (42%) presented with elevated SPAP defined as above 30 mmHg. If we select patients with more severe PHT using a MAP/MPAP ratio < 2, there were only 16 patients, all experienced difficult separation from CPB, 3 died (18.7% mortality) and half required vasoactive support for more than 24 hours after the procedure.

For this reason, it is relatively clear that the presence of PHT before the operation or appearing during or after it will have an impact on survival and mostly through its effect on right ventricular function. The next question is: how can we prevent or treat PHT and its consequence, RV failure?

## 4. PHARMACOLOGIC AND NON-PHARMACOLOGIC APPROACHES IN THE TREATMENT AND PREVENTION OF PULMONARY HYPERTENSION IN CARDIAC SURGERY

The choice of the appropriate therapy should be based on evidence-based medicine. A MEDLINE search was performed using the key words ‘randomized controlled trial’ (RCT), ‘humans’, ‘adults’, ‘English’ and ‘PHT’. Articles related to cardiac surgery were then selected and classified according to the levels of evidence proposed by Sackett [42] for evidence-based medical practice. Using this strategy, a total of 10 articles were retrieved. In addition, the Consort statement group has developed guidelines to assess the quality of randomized controlled clinical trials [43]. These studies are summarized in Table **3**.

### 4.1. Treatment of Pulmonary Hypertension

#### 4.1.1. Pharmacological

The agents studied were: inhaled prostacyclin (PGI_2_), nitric oxide (NO) and intravenous vasodilators such as prostacyclin E1 (PGE1), nitroglycerin (NTG), nitroprusside (NTP), milrinone, enoximone and dobutamine. One large RCT compared heparinise to protamine and explored as a secondary end-point the prevention of PHT from protamine administration [44]. Most of the studies reviewed included a small number of patients and their primary end-points were hemodynamic changes.

In the most recent trial, Fattouch *et al.* [45] studied patients with PHT (*n*=58) undergoing MVR for mitral stenosis. Inhaled PGI_2_ (iPGI_2_) and iNO were compared to conventional intravenous vasodilators. The inhaled drugs were given just before the end of CPB. Significant reductions in PHT indices as well as increase in cardiac output (CO) and in RV ejection fraction were observed in both inhaled groups compared to conventional treatment. In addition, in both inhaled groups, separation from CPB was easier, the amount of vasoactive drugs administered was smaller and the duration of stay in the ICU and hospital was shorter. The same group also compared the same three strategies in the treatment of PHT after MVR upon arrival in the intensive care unit (ICU) [46]. Inhalation of PGI_2_ was associated with a reduction in PVR and an increase in stroke volume. Inhaled NO reduced PVR but did not increase stroke volume, and NTP was associated with a reduction in systemic arterial pressure and systemic vascular resistance.

The administration of protamine can be associated with severe PHT followed by RV failure. This condition requires immediate treatment. In a study of CABG patients (*n*=3800), Ocal *et al. *[14] compared two therapeutic approaches in the treatment of the protamine reaction observed in 68 patients (1.8%). One group received iPGI_2_ and the other intravenous NTG in addition to standard vasoactive agents. The iPGI_2_ group showed improved hemodynamics and only 14 patients (39%) had to return on CPB compared to all 30 patients (100%) in the NTG group. A tendency for shorter length of stay in the ICU and reduced mortality was observed in the iPGI_2_ group, but the numbers were too small to be statistically significant.

To avoid protamine reaction, heparinise I, a heparin degrading enzyme, was compared in a multicentered randomized controlled trial [44]. However, the results of the trial were negative and heparinise I was not associated with any reduction in the intervention to treat PHT or any reduction in bleeding.

Solina *et al.* explore the dose-responsiveness of iNO given on termination of CPB at 10, 20, 30 and 40 ppm compared to intravenous milrinone [47]. Nitric oxide was associated with a reduction in PVR with a maximum dose of 10 ppm. No significant difference in reduction of PVR or inotropic requirement was observed compared to milrinone. The same authors compared NO 20 ppm and 40 ppm to milrinone in patients with PVR above 125 after cardiac surgery [48]. The drugs were started after CPB and for 24 hours in the intensive care unit. Higher systemic arterial pressure was observed in the 20 ppm group and higher RVEF were obtained in the 40 ppm NO group. The milrinone group required significantly more phenylephrine and tended to have higher heart rate than either of the NO groups in the ICU.

In patients with CO below 2 L/min/m² and PAOP > 10 mmHg, Feneck compared milrinone to dobutamine in 120 patients [49]. In a subset of patients with PHT defined as (PVR >200 dyne sec cm5; MPAP > 25 mmHg), milrinone had a similar effect to dobutamine on the reduction of PVR and increase in cardiac index (CI). The PAOP and systemic vascular resistance (SVR) were more reduced by milrinone.

Schmid *et al. *[50] compared three approaches (iNO *vs* PGE1 *vs* NTG) in a crossover study; these were used to treat PHT after cardiac surgery in 14 patients. Only stable patients were included in the study, which limits the application of the results. Inhaled NO decreased PVR without reducing SVR, did not change coronary perfusion pressure of the right coronary pressure and increased oxygen transport.

Finally, Hachenberger *et al. *[51] explored the role of enoximone compared to NTG and dobutamine, given after induction of anesthesia and then restarted before the end of CPB. Only enoximone was associated with a decrease in MPAP and PVR.

In our practice at the Montreal Heart Institute, we regularly use iPGI_2_ [52, 53] and inhaled milrinone [54, 55] in the presence of pulmonary hypertension and RV dysfunction before and after cardiac surgery. Inhaled NO and oral sildenafil are used in refractory cases in the ICU.

#### 4.1.2. Non-Pharmacological Approach

The non-pharmacological approach to the treatment of PHT will be directed to the cause or the consequence of PHT, as illustrated in Fig. (**5**). In the presence of PHT secondary to LV failure, intra-aortic balloon counterpulsation will facilitate recovery of LV dysfunction. If prosthetic valve dysfunction is present after CPB, then return on CPB and correction of the problem will be the treatment of choice. The correction of hypoxia, hypercapnia and surgical thrombo-embolectomy (when surgically indicated) can help control PHT. In patients with elevated intrathoracic pressure from accumulated air or blood, chest drainage will be the solution. However, in some patients undergoing long procedures and long CPB duration, chest closure can be associated with hemodynamic instability. This is a sort of “thoracic compartment” syndrome. In these situations, the chest temporarily can be left open to reduce the surrounding pressures. Finally, pulmonary artery balloon pump, RV assist device (RVAD) or cavopulmonary diversion have been described as potential treatments for severe RV dysfunction [10].

### 4.2. Treatment of Right Ventricular Failure

We summarize our approach to the treatment of RV failure in Fig. (**9**). Right ventricular function is evaluated visually, using the RV pressure waveform and TEE. Once RVOTO is ruled out, the etiology of RV systolic dysfunction is divided in two categories. If ischemia is suspected to contribute to RV failure, then both the medical and the surgical treatment will be oriented toward the promotion of RV perfusion. In a non-ischemic etiology is suspected, the medical and surgical treatment will be oriented toward an increase in contractility (inotropes) and a reduction in RV afterload (iNO, iPGI_2_, inhaled milrinone).

## 5. PREVENTION OF PULMONARY HYPERTENSION

### 5.1. Pharmacological Approach

The prevention of PHT and its consequences could represent a promising strategy to prevent RV failure. However, very few studies have addressed this issue. One of the potential targets could be the prevention of the pulmonary reperfusion syndrome. In that regard, our group has demonstrated in an animal model that iPGI2 [56] and inhaled milrinone [54] could prevent endothelial dysfunction induced by CPB. Hache *et al. *[53] conducted a pilot RCT in patients with preoperative PHT and demonstrated that iPGI2 was superior to placebo in reducing PHT. Furthermore, in patients who received iPGI2, the amount of vasoactive support was reduced. 

We have completed a randomized controlled trial on the use of inhaled milrinone administered before CPB in 21 patients. There were 8 males and 13 females of a mean age of 70±6.3 years and a mean Parsonnet Score of 32±9. All procedures were valvular surgeries, 14 of which were complex surgeries and 5 reoperations. Mean systolic pulmonary artery pressures (SPAP) were reduced in the inhaled milrinone group from 66±20 mmHg (pre-CPB) to 46±20 mmHg (after CPB) (*p*<0.001). No changes in SPAP were observed in the control group and no differences in systemic arterial pressure between the groups were observed. We also published our preliminary experience in the use of inhaled milrinone involving 70 high risk patients (Parsonnet Score of 27±14) [27, 55]. Compared with a control group with similar baseline characteristics, we observed that the administration of inhaled milrinone prior to CPB (*n*=30) was associated with a lesser rate of re-initiation of CPB (9 *vs* 1; *p*=0.021) in this very high risk group. We also observed postoperatively lower pulmonary artery pressures in the pre-CPB group. Further studies will be required to determine the efficacy of this approach.

### 5.2. Non-Pharmacological Approach

The selection of the type and size of an aortic prosthetic valve could be a very important strategy, because it has been shown that, if the effective orifice area (EOA) of the aortic valve is too small in relation to body size, the so-called PPM, the intraoperative and long-term mortality, is increased [57-64]. Hence, anticipatory strategies aiming at the prevention of PPM, such as the implantation of a better performing prosthesis (i.e. stentless bioprosthesis, new generation bileaflet mechanical valve, new generation supra-annular stented bioprosthetic valve) or the enlargement of the aortic root to accommodate a larger prosthesis could contribute to reduce PHT after cardiac surgery and facilitate the separation from CPB. On the other hand, some of the alternative options that can be used to prevent PPM are complex and may increase the risk of DSB by prolonging the duration of the surgical procedure and thus CPB time. As a consequence, in some cases, the drawbacks of using alternative procedures may overcome the benefits of avoiding PPM. It is therefore essential to establish accurate criteria to better assess the risk-benefit ratio with respect to the prevention of PPM. For mitral valve PPM, the best way would be to repair rather than replace the mitral valve. However, mitral valve repair cannot be provided to a significant number of patients and the options are more limited than for aortic valve replacement [16].

## CONCLUSION

In summary, PHT in cardiac surgery is an important variable in cardiac surgery. It should be diagnosed before cardiac surgery and the impact of PHT on RV function determined. Future trials should address the role of preemptive reduction in the severity of PHT before cardiac surgery and their impact on post-operative outcome.

## Figures and Tables

**Fig. (1):**
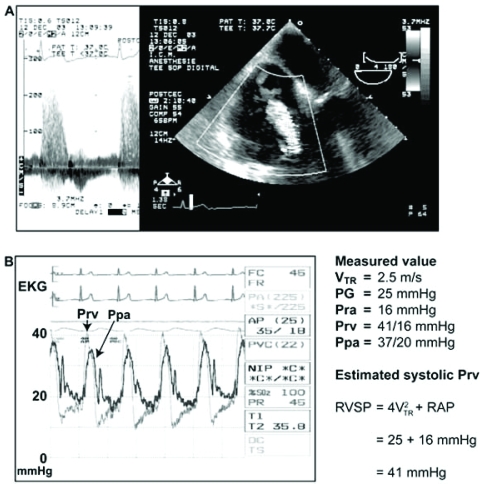
(A) Estimation of right ventricular systolic pressure (systolic Prv or RVSP) using the pressure gradient (PG) obtained from tricuspid regurgitation (TR) and right atrial pressure (Pra or RAP). (B) Note that the RVSP is higher than the systolic pulmonary artery pressure (Ppa) due to a small gradient across the pulmonic valve (EKG: electrocardiogram, V: velocity). With permission from Denault *et al*. [2] (Chapter 5, p.111).

**Fig. (5):**
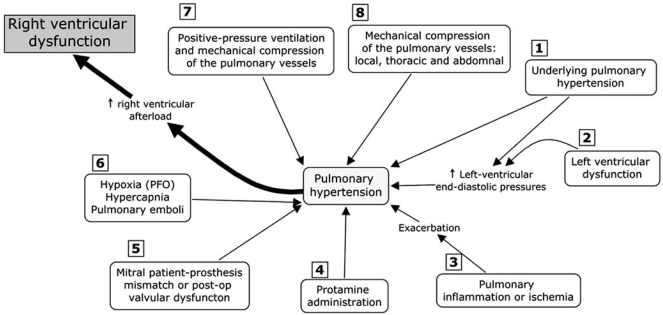
The most common mechanisms that could induce pulmonary hypertension in cardiac surgery. (See Section 2.1 for details) (PFO: patent foramen ovale).

**Fig. (9):**
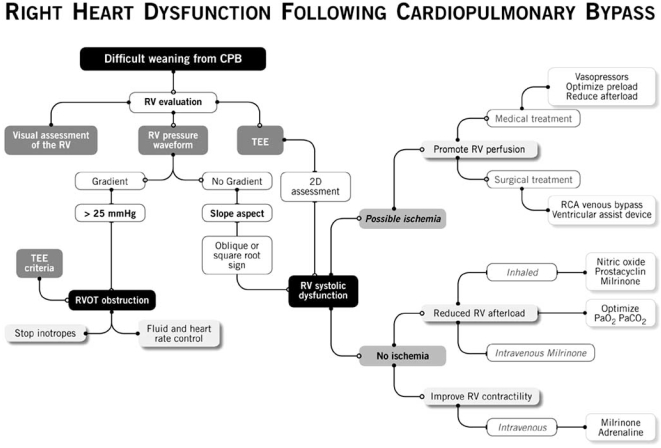
Proposed approach in the treatment of right ventricular (RV) dysfunction. (RCA: right coronary artery, RVOT: right ventricular outflow tract, TEE: transesophageal echocardiography) (Presented by Dr. Y. Lamarche at the 2006 CCS meeting in Vancouver).

**Table 1.** Definitions of Pulmonary Hypertension Used in Clinical Research

Hemodynamic Parameter [1]	Normal Value	Abnormal Value
Systolic pulmonary artery pressure (SPAP)	15-30 mmHg	> 30 or ≥ 40 mmHg
Mean pulmonary artery pressure (MPAP)	9-16 mmHg	Moderate: > 18 mmHg Significant: > 25 mmHg Exercise-induced: > 30 mmHg
Pulmonary vascular resistance (PVR) = (MPAP – PAOP) X 80/CO	60-120 dyn·s·cm^-5^ 1.1-1.4 Wood unit	Mild: > 125 dyn s cm^-5^ Moderate: > 200-300 dyn s cm^-5^ Severe: > 600dyn s cm^-5^
Indexed pulmonary vascular resistance (PVRI) = (MPAP – PAOP) X 80/CI	250-340 dyn·s·cm^-5^·m^-2^	
Pulmonary to systemic vascular resistance index (PVRI/SVRI) X 100%	< 10%	
Trans-pulmonary gradient (MPAP – PAOP)	< 14 mmHg	
Mean pulmonary to systemic pressure ratio (MPAP/MAP) X 100%	< 25%	Moderate: 33-50% Severe: > 50%
Mean systemic to pulmonary pressure ratio (MAP/MPAP) X 100%	> 4	< 4 [3]

CO: cardiac output, CI: cardiac index, PAOP: pulmonary artery occlusion pressure.

**Table 2.** Prognostic Value of Right Ventricular Function in Cardiac Surgery (Selected Studies)

Study	Population	Study Design	RV Dysfunction	Results
Reitchert *et al*. [65]	Unstable post-operative patients	Prospective *n*=60	RVFAC < 35%	RV dysfunction associated with high mortality rates
Pinzani *et al*. [32]	Mitral and combined mitro-aortic surgery	Retrospective *n*=382	Clinical definition	Post-operative RV failure is the strongest predictor of postoperative mortality
Cullen *et al*. [66]	Tetralogy of Fallot	Prospective *n*=35	Restrictive RV physiology	Restrictive physiology predicts longer intensive care unit stay post repair and lower cardiac output
Gatzoulis *et al*. [67]	Tetralogy of Fallot	Prospective *n*=41	Restrictive RV physiology	Restrictive physiology predicts smaller RV and better exercise tolerance
Kromos *et al*. [68]	LVAD and RV failure	Retrospective *n*=31	Clinical mean RVEF = 11.8%	Preoperative clinical factors such as fever, pulmonary edema, and intraoperative blood transfusions were associated with RVAD need
Hosenpud *et al*. [69]	Heart Transplantation	Retrospective International Society for Heart & Lung transplantation *n*=69,205	RV failure associated with circulatory failure	RV failure accounts for up to 20% of early deaths
Oehiai *et al*. [70]	LVAD	Retrospective *n*=245	RV failure requiring RVAD	23 patients (9%) required RVAD. The need for circulatory support, female gender, and non-ischemic etiology were predictors of RVAD need.
Maslow *et al*. [34]	CAD undergoing coronary bypass surgery with LVEF < 25%	Retrospective *n*=41	RVFAC < 35%	RV dysfunction is associated with decreased long term survival
Therrien *et al*. [71]	Tetralogy of Fallot	Prospective *n*=17	RV remodeling	Severe RV dilatation (RVEDV ≥ >170 ml/m2 or RVESV >85 ml/m2) associated with incomplete RV remodeling
Webb *et al*. [72, 73]	Atrial septal defect	Retrospective series	RV remodeling	Older age at repair and abnormal RV myocardial relaxation were associated with incomplete RV remodeling
Denault *et al.* [74]	Patients undergoing bypass surgery	Retrospective and prospective *n*=800	Dynamic obstruction of RVOT (Gd > 25 mmHg)	Incidence: 4%, dynamic obstruction of RVOT was associated with a higher incidence of difficult weaning from bypass
Haddad *et al*.[35]	High risk valvular surgery	Prospective *n*=50	RVFAC < 32% or RVMPI > 0.50	Preoperative RV dysfunction was associated with a higher incidence of post-operative circulatory failure

CAD: coronary artery disease, Gd: gradient, LV: left ventricular, LVAD: left ventricular assist device, RV: right ventricular, RVAD: right ventricular assist device, RVES: right ventricular end-systolic volume, RVED: right ventricular end-diastolic volume, RVEF: right ventricular ejection fraction, RVFAC: right ventricular fractional area change, RVMPI: right ventricular myocardial performance index, RVOT: right ventricular outflow tract obstruction. Based on [25].

**Table 3.** Randomized Controlled Trial in the Treatment of Pulmonary Hypertension in Adult Cardiac Surgery

	Author	Country	Date	Agents Used	Design	N	Inclusion criteria	Primary End-Point	Level of Evidence
1	Fattouch *et al*. [45]	Italy	2006	iPGI2 *vs* iNO vs intravenous vasodilators	RCT Unicenter	58	MVR + PHT before the end of CPB	Hemodynamic	A1b
2	Ocal *et al*. [14]	Turkey	2005	iPGI2 *vs* NTG	RCT Multicenter	68	CABG with protamine reaction after CPB	Hemodynamic	A1b
3	Stafford *et al*. [44]	USA NC	2005	Heparinase *vs* protamine	Non-inferiority clinical trial design Multicenter	167	CABG on + off pump after CPB	Bleeding	A1b
4	Fattouch *et al*. [46]	Italy	2005	iPGI2 *vs* iNO *vs* intravenous vasodilators	RCT Unicenter	58	MVR + PHT in the intensive care unit	Hemodynamic	A1b
5	Hache *et al*. [53]	Canada	2003	iPGI2 *vs* placebo	RCT Unicenter	20	PHT before CPB	Hemodynamic	A1b
6	Solina *et al*. [47]	USA	2001	iNO *vs* milrinone	RCT Unicenter	62	PHT after surgery	Hemodynamic	B
7	Feneck *et al*. [49]	UK	2001	Milrinone *vs* dobutamine	RCT Multicenter	120	CO < 2 L/min/m² et PAOP > 10 mmHg after cardiac surgery	Hemodynamic	A1b
8	Solina *et al*. [48]	USA	2000	iNO *vs* milrinone	RCT Unicenter	45	PHT after surgery	Hemodynamic	A1b
9	Schmid *et al*. [50]	Switzerland	1999	iNO *vs* NTG *vs* PGE1	Crossover Unicenter	14	PHT after surgery	Hemodynamic	B
10	Hachenberg *et al*. [51]	Germany	1997	Enoximone *vs* dobutamine+NTG	RCT Unicenter	20	HTP in MVR before and after surgery	Hemodynamic	A1b

CABG: coronary artery bypass graft, CO: cardiac output, CPB: cardiopulmonary bypass, iNO: inhaled nitric oxide, iPGI2: inhaled prostacyclin, MVR: mitral valve replacement, NO: nitric oxide, NTG: nitroglycerin, OR: operating room, PAOP: pulmonary artery occlusion pressure, PGE1: prostaglandin E1, PGI2: prostacyclin, PHT: pulmonary hypertension, RCT: randomized controlled trial, UK: United Kingdom, USA: United States of America.

## References

[1] Gomez CM, Palazzo MG (1998). Pulmonary artery catheterization in anaesthesia and intensive care. Br J Anaesth.

[2] Denault AY, Couture P, Tardif JC, Buithieu J (2005). Transesophageal echocardiography multimedia manual: a perioperative transdisciplinary approach. Marcel Dekker.

[3] Robitaille A, Denault AY, Couture P (2006). Importance of relative pulmonary hypertension in cardiac surgery: the mean systemic-to-pulmonary artery pressure ratio. J Cardiothorac Vasc Anesth.

[4] Therrien J, Dore A, Gersony W (2001). CCS Consensus Conference 2001 update: recommendations for the management of adults with congenital heart disease. Part I. Can J Cardiol.

[5] Therrien J, Gatzoulis M, Graham T (2001). Canadian Cardiovascular Society Consensus Conference 2001 update: Recommendations for the Management of Adults with Congenital Heart Disease--Part II. Can J Cardiol.

[6] Simonneau G, Galie N, Rubin LJ (2004). Clinical classification of pulmonary hypertension. J Am Coll Cardiol.

[7] Denault AY, Ferraro P, Couture P (2003). Transesophageal echocardiography monitoring in the intensive care department: the management of hemodynamic instability secondary to thoracic tamponade after single lung transplantation. J Am Soc Echocardiogr.

[8] Bakhtiary F, Schiemann M, Dzemali O (2007). Impact of patient-prosthesis mismatch and aortic valve design on coronary flow reserve after aortic valve replacement. J Am Coll Cardiol.

[9] Lesage AM, Tsuchioka H, Young WG Jr, Sealy WC (1966). Pathogenesis of pulmonary damage during extracorporeal perfusion. Arch Surg.

[10] Kaul TK, Fields BL (2000). Postoperative acute refractory right ventricular failure: incidence, pathogenesis, management and prognosis. Cardiovasc Surg.

[11] Downing SW, Edmunds LH Jr (1992). Release of vasoactive substances during cardiopulmonary bypass. Ann Thorac Surg.

[12] Wan S, LeClerc JL, Vincent JL (1997). Inflammatory response to cardiopulmonary bypass: mechanisms involved and possible therapeutic strategies. Chest.

[13] Asimakopoulos G, Smith PL, Ratnatunga CP, Taylor KM (1999). Lung injury and acute respiratory distress syndrome after cardiopulmonary bypass. Ann Thorac Surg.

[14] Ocal A, Kiris I, Erdinc M, Peker O, Yavuz T, Ibrisim E (2005). Efficiency of prostacyclin in the treatment of protamine-mediated right ventricular failure and acute pulmonary hypertension. Tohoku J Exp Med.

[15] Viaro F, Dalio MB, Evora PR (2002). Catastrophic cardiovascular adverse reactions to protamine are nitric oxide/cyclic guanosine monophosphate dependent and endothelium mediated: should methylene blue be the treatment of choice?. Chest.

[16] Magne J, Mathieu P, Dumesnil JG (2007). Impact of prosthesis-patient mismatch on survival after mitral valve replacement. Circulation.

[17] Sukernik MR, Mets B, Bennett-Guerrero E (2001). Patent foramen ovale and its significance in the perioperative period. Anesth Analg.

[18] Fischer LG, Van AH, Burkle H (2003). Management of pulmonary hypertension: physiological and pharmacological considerations for anesthesiologists. Anesth Analg.

[19] Humbert M, Sitbon O, Simonneau G (2004). Treatment of pulmonary arterial hypertension. N Engl J Med.

[20] McLaughlin VV, McGoon MD (2006). Pulmonary arterial hypertension. Circulation.

[21] D'Alonzo GE, Barst RJ, Ayres SM (1991). Survival in patients with primary pulmonary hypertension. Results from a national prospective registry. Ann Intern Med.

[22] Yeo TC, Dujardin KS, Tei C, Mahoney DW, McGoon MD, Seward JB (1998). Value of a Doppler-derived index combining systolic and diastolic time intervals in predicting outcome in primary pulmonary hypertension. Am J Cardiol.

[23] Ramakrishna G, Sprung J, Ravi BS, Chandrasekaran K, McGoon MD (2005). Impact of pulmonary hypertension on the outcomes of noncardiac surgery: predictors of perioperative morbidity and mortality. J Am Coll Cardiol.

[24] Voelkel NF, Quaife RA, Leinwand LA (2006). Right ventricular function and failure: report of a National Heart, Lung, and Blood Institute working group on cellular and molecular mechanisms of right heart failure. Circulation.

[25] Haddad F, Couture P, Tousignant C, Denault AY (2009). The right ventricle in cardiac surgery, a perioperative perspective: II. Pathophysiology, clinical importance, and management. Anesth Analg.

[26] Chin KM, Kim NH, Rubin LJ (2005). The right ventricle in pulmonary hypertension. Coron Artery Dis.

[27] Bernstein AD, Parsonnet V (2000). Bedside estimation of risk as an aid for decision-making in cardiac surgery. Ann Thorac Surg.

[28] Nashef SA, Roques F, Hammill BG (2002). Validation of European System for Cardiac Operative Risk Evaluation (EuroSCORE) in North American cardiac surgery. Eur J Cardiothorac Surg.

[29] Shroyer AL, Coombs LP, Peterson ED (2003). The Society of Thoracic Surgeons: 30-day operative mortality and morbidity risk models. Ann Thorac Surg.

[30] Ambler G, Omar RZ, Royston P, Kinsman R, Keogh BE, Taylor KM (2005). Generic, simple risk stratification model for heart valve surgery. Circulation.

[31] Boldt J, Zickmann B, Ballesteros M, Dapper F, Hempelmann G (1992). Right ventricular function in patients with aortic stenosis undergoing aortic valve replacement. J Cardiothorac Vasc Anesth.

[32] Pinzani A, de Gevigney G, Pinzani V, Ninet J, Milon H, Delahaye JP (1993). Pre- and postoperative right cardiac insufficiency in patients with mitral or mitral-aortic valve diseases. Arch Mal Coeur Vaiss.

[33] Wencker D, Borer JS, Hochreiter C (2000). Preoperative predictors of late postoperative outcome among patients with nonischemic mitral regurgitation with 'high risk' descriptors and comparison with unoperated patients. Cardiology.

[34] Maslow AD, Regan MM, Panzica P, Heindel S, Mashikian J, Comunale ME (2002). Precardiopulmonary bypass right ventricular function is associated with poor outcome after coronary artery bypass grafting in patients with severe left ventricular systolic dysfunction. Anesth Analg.

[35] Haddad F, Denault AY, Couture P (2007). Right ventricular myocardial performance index predicts perioperative mortality or circulatory failure in high-risk valvular surgery. J Am Soc Echocardiogr.

[36] Davila-Roman VG, Waggoner AD, Hopkins WE, Barzilai B (1995). Right ventricular dysfunction in low output syndrome after cardiac operations: assessment by transesophageal echocardiography. Ann Thorac Surg.

[37] Tuman KJ, McCarthy RJ, March RJ, Najafi H, Ivankovich AD (1992). Morbidity and duration of ICU stay after cardiac surgery. A model for preoperative risk assessment. Chest.

[38] Tremblay NA, Hardy JF, Perrault J, Carrier M (1993). A simple classification of the risk in cardiac surgery: the first decade. Can J Anaesth.

[39] Reich DL, Bodian CA, Krol M, Kuroda M, Osinski T, Thys DM (1999). Intraoperative hemodynamic predictors of mortality, stroke, and myocardial infarction after coronary artery bypass surgery. Anesth Analg.

[40] Malouf JF, Enriquez-Sarano M, Pellikka PA (2002). Severe pulmonary hypertension in patients with severe aortic valve stenosis: clinical profile and prognostic implications. J Am Coll Cardiol.

[41] Nilsson J, Algotsson L, Hoglund P, Luhrs C, Brandt J (2006). Comparison of 19 pre-operative risk stratification models in open-heart surgery. Eur Heart J.

[42] Sackett DL (1989). Rules of evidence and clinical recommendations on the use of antithrombotic agents. Chest.

[43] Moher D, Schulz KF, Altman DG (2001). The CONSORT statement: revised recommendations for improving the quality of reports of parallel-group randomised trials. Lancet.

[44] Stafford-Smith M, Lefrak EA, Qazi AG (2005). Efficacy and safety of heparinase I versus protamine in patients undergoing coronary artery bypass grafting with and without cardiopulmonary bypass. Anesthesiology.

[45] Fattouch K, Sbraga F, Bianco G (2005). Inhaled prostacyclin, nitric oxide, and nitroprusside in pulmonary hypertension after mitral valve replacement. J Card Surg.

[46] Fattouch K, Sbraga F, Sampognaro R (2006). Treatment of pulmonary hypertension in patients undergoing cardiac surgery with cardiopulmonary bypass: a randomized, prospective, double-blind study. J Cardiovasc Med (Hagerstown ).

[47] Solina AR, Ginsberg SH, Papp D (2001). Dose response to nitric oxide in adult cardiac surgery patients. J Clin Anesth.

[48] Solina A, Papp D, Ginsberg S (2000). A comparison of inhaled nitric oxide and milrinone for the treatment of pulmonary hypertension in adult cardiac surgery patients. J Cardiothorac Vasc Anesth.

[49] Feneck RO, Sherry KM, Withington PS, Oduro-Dominah A (2001). Comparison of the hemodynamic effects of milrinone with dobutamine in patients after cardiac surgery. J Cardiothorac Vasc Anesth.

[50] Schmid ER, Burki C, Engel MH, Schmidlin D, Tornic M, Seifert B (1999). Inhaled nitric oxide versus intravenous vasodilators in severe pulmonary hypertension after cardiac surgery. Anesth Analg.

[51] Hachenberg T, Mollhoff T, Holst D, Hammel D, Brussel T (1997). Cardiopulmonary effects of enoximone or dobutamine and nitroglycerin on mitral valve regurgitation and pulmonary venous hypertension. J Cardiothorac Vasc Anesth.

[52] Hache M, Denault AY, Belisle S (2001). Inhaled prostacyclin (PGI2) is an effective addition to the treatment of pulmonary hypertension and hypoxia in the operating room and intensive care unit. Can J Anaesth.

[53] Hache M, Denault AY, Belisle S (2003). Inhaled epoprostenol (prostacyclin) and pulmonary hypertension before cardiac surgery. J Thorac Cardiovasc Surg.

[54] Lamarche Y, Malo O, Thorin E (2005). Inhaled but not intravenous milrinone prevents pulmonary endothelial dysfunction after cardiopulmonary bypass. J Thorac Cardiovasc Surg.

[55] Lamarche Y, Perrault LP, Maltais S, Tetreault K, Lambert J, Denault AY (2007). Preliminary experience with inhaled milrinone in cardiac surgery. Eur J Cardiothorac Surg.

[56] Fortier S, DeMaria RG, Lamarche Y (2004). Inhaled prostacyclin reduces cardiopulmonary bypass-induced pulmonary endothelial dysfunction via increased cyclic adenosine monophosphate levels. J Thorac Cardiovasc Surg.

[57] Milano AD, Blanzola C, Mecozzi G (2001). Hemodynamic performance of stented and stentless aortic bioprostheses. Ann Thorac Surg.

[58] Rao V, Jamieson WR, Ivanov J, Armstrong S, David TE (2000). Prosthesis-patient mismatch affects survival after aortic valve replacement. Circulation.

[59] Pibarot P, Dumesnil JG (2000). Hemodynamic and clinical impact of prosthesis-patient mismatch in the aortic valve position and its prevention. J Am Coll Cardiol.

[60] Blais C, Dumesnil JG, Baillot R, Simard S, Doyle D, Pibarot P (2003). Impact of valve prosthesis-patient mismatch on short-term mortality after aortic valve replacement. Circulation.

[61] Ruel M, Rubens FD, Masters RG (2004). Late incidence and predictors of persistent or recurrent heart failure in patients with aortic prosthetic valves. J Thorac Cardiovasc Surg.

[62] Pibarot P, Dumesnil JG (2006). Prosthesis-patient mismatch: definition, clinical impact, and prevention. Heart.

[63] Tasca G, Mhagna Z, Perotti S (2006). Impact of prosthesis-patient mismatch on cardiac events and midterm mortality after aortic valve replacement in patients with pure aortic stenosis. Circulation.

[64] Kulik A, Burwash IG, Kapila V, Mesana TG, Ruel M (2006). Long-term outcomes after valve replacement for low-gradient aortic stenosis: impact of prosthesis-patient mismatch. Circulation.

[65] Reichert CL, Visser CA, van den Brink RB (1992). Prognostic value of biventricular function in hypotensive patients after cardiac surgery as assessed by transesophageal echocardiography. J Cardiothorac Vasc Anesth.

[66] Cullen S, Shore D, Redington A (1995). Characterization of right ventricular diastolic performance after complete repair of tetralogy of Fallot. Restrictive physiology predicts slow postoperative recovery. Circulation.

[67] Gatzoulis MA, Till JA, Somerville J, Redington AN (1995). Mechanoelectrical interaction in tetralogy of Fallot. QRS prolongation relates to right ventricular size and predicts malignant ventricular arrhythmias and sudden death. Circulation.

[68] Kormos RL, Gasior TA, Kawai A (1996). Transplant candidate's clinical status rather than right ventricular function defines need for univentricular versus biventricular support. J Thorac Cardiovasc Surg.

[69] Hosenpud JD, Bennett LE, Keck BM, Boucek MM, Novick RJ (2000). The Registry of the International Society for Heart and Lung Transplantation: seventeenth official report-2000. J Heart Lung Transplant.

[70] Ochiai Y, McCarthy PM, Smedira NG (2002). Predictors of severe right ventricular failure after implantable left ventricular assist device insertion: analysis of 245 patients. Circulation.

[71] Therrien J, Provost Y, Merchant N, Williams W, Colman J, Webb G (2005). Optimal timing for pulmonary valve replacement in adults after tetralogy of Fallot repair. Am J Cardiol.

[72] Davlouros PA, Niwa K, Webb G, Gatzoulis MA (2006). The right ventricle in congenital heart disease. Heart.

[73] Webb JG, Chandavimol M, Thompson CR (2006). Percutaneous aortic valve implantation retrograde from the femoral artery. Circulation.

[74] Denault AY, Chaput M, Couture P, Hebert Y, Haddad F, Tardif JC (2006). Dynamic right ventricular outflow tract obstruction in cardiac surgery. J Thorac Cardiovasc Surg.

